# Intrahepatic Cholestasis of Pregnancy Leading to Severe Vitamin K Deficiency and Coagulopathy

**DOI:** 10.1155/2017/5646247

**Published:** 2017-06-07

**Authors:** Maria Maldonado, Ali Alhousseini, Michael Awadalla, Jay Idler, Robert Welch, Karoline Puder, Manasi Patwardhan, Bernard Gonik

**Affiliations:** Department of Obstetrics and Gynecology, Wayne State University School of Medicine, Detroit, MI, USA

## Abstract

Intrahepatic cholestasis of pregnancy is seldom associated with significant vitamin K deficiency. We report a case of a 16-year-old primigravid patient at 24 weeks and 3 days of gestation who presented with pruritus, hematuria, and preterm labor. Laboratory work-up showed severe coagulopathy with Prothrombin Time (PT) of 117.8 seconds, International Normalized Ratio (INR) of 10.34, and elevated transaminases suggestive of intrahepatic cholestasis of pregnancy. Her serum vitamin K level was undetectable (<0.1 nMol/L). Initial therapy consisted of intramuscular replacement of vitamin K and administration of fresh frozen plasma. Her hematuria and preterm labor resolved and she was discharged. She presented in active labor and delivered at 27 weeks and 1 day. Her bile acids (93 *μ*/L) and INR (2.32) had worsened. She delivered a male infant, 1150 grams with Apgar scores 7 and 9. The newborn received 0.5 mg of intramuscular vitamin K shortly after delivery but went on to develop bilateral grade III intraventricular hemorrhages by day 5. Intrahepatic cholestasis in pregnancy and nutrition issues were identified as the main risk factors for the severe coagulopathy of this patient. This case underlines the importance of evaluation of possible severe coagulopathy in patients with intrahepatic cholestasis of pregnancy in order to avoid serious maternal or fetal adverse outcomes.

## 1. Introduction

Obstetric hemorrhage is the leading direct cause of both maternal mortality and near-miss events [1]. Intrahepatic cholestasis of pregnancy is seldom associated with significant vitamin K deficiency and severe coagulopathy. Usually, nutritional deficiencies, medication usage, and liver disease lead to this disorder [2]. The fetus and the newborn are dependent on maternally derived vitamin K facilitated diffusion transport across the placenta for coagulation stability as well [3, 4]. We report a patient presenting with pruritus, preterm labor, and hematuria, finally diagnosed with intrahepatic cholestasis of pregnancy (ICP) complicated with pathologic vitamin K deficiency.

## 2. Case Report

A 16-year-old primigravid woman presented to labor and delivery at 24 weeks and 3 days of gestational age with no prenatal care. She had severe, generalized pruritus, uterine contractions, and hematuria. She noted gingival bleeding over the prior year. On examination, her body mass index was 18.6 kg/m^2^ and her vital signs were unremarkable. She had no rashes, petechiae, ecchymosis, or hematomas. She was having uterine contractions and progressive cervical change. Betamethasone, magnesium sulfate, and indomethacin were initiated. Initial laboratory evaluation showed severe coagulopathy with Prothrombin Time (PT) of 117.8 seconds, International Normalized Ratio (INR) of 10.34, and elevated transaminases (Table 1). Vitamin K level was undetectable (<0.1 nMol/L) and factor VII activity was 3% (normal range 82–128%). The patient was given 10 mg of vitamin K intramuscularly and 4 units of fresh frozen plasma. Repeat laboratory studies 12 hours later showed that vitamin K level, PT, PTT, and INR had normalized. Preterm labor arrested at cervical dilatation of 2 centimeters and the hematuria resolved. Ultrasound demonstrated an appropriately grown fetus without any abnormalities and normal amniotic fluid volume. On hospital day #6 the patient was asymptomatic and discharged home. She returned five days later to our outpatient clinic. She continued to have generalized pruritus. Bile acid results were pending and were later reported to be 29 micromole/L (normal range 0–10 micromole/L). The patient was prescribed supplemental oral vitamin K therapy.

She was readmitted at 26 weeks and 4 days with recurrence of premature labor symptoms. Her INR, PT, and PTT were again elevated at 2.32, 25.2, and 47.9 seconds, respectively. Two units of fresh frozen plasma were administered. The patient received a rescue dose of betamethasone. Magnesium sulfate and indomethacin were initiated. Premature labor symptoms abated. Repeat total bile acids were 93 micromole/L. She was started on oral Ursodeoxycholic acid 300 mg twice daily. At 27 weeks and 1 day of gestation she delivered a male infant weighing 1150 grams with Apgar scores of 7 and 9 (1-minute and 5-minute, resp.). Estimated blood loss at delivery was 200 mL.

The newborn showed no signs of bruising. Vitamin K 0.5 mg was administered intramuscularly. No coagulation studies or vitamin K levels were obtained. The neonatal course was complicated by respiratory distress requiring intubation. On the fifth day neonatal head imaging showed bilateral grade 3 intraventricular hemorrhage (IVH). There were no other sites of active fetal bleeding. The newborn spent 50 days in the neonatal intensive care unit. Repeat head ultrasound at two months showed resolution of the IVH. Ambulatory clinic follow-up at age 4 months showed that the infant was meeting normal milestones.

## 3. Discussion

Vitamin K deficiency in pregnancy is a rare but very serious event. Having a high suspicion for vitamin K deficiency in patients who have similar presentation or laboratory work-up is an important teaching point. To date, there is no report of vitamin K deficiency and coagulopathy associated with cholestasis of pregnancy in the literature. A Medline search only identified anecdotal and conflicting reports of increased postpartum hemorrhage and fetal intraventricular bleeding with suspected cholestasis of pregnancy cases [5, 6]. These relatively old case studies lack confirmatory laboratory analyses for bile acids and vitamin K levels.

Cholestasis in pregnancy causes reduced enterohepatic circulation of bile acids and a subsequent reduction in the absorption of fat soluble vitamins (D, E, A, K) from the terminal ileum. Fetal vitamin K is derived from the mother through transplacental passage. At birth, activities of the vitamin K dependent factors II, VII, IX, and X and the concentrations of the contact factors XI and XII are reduced to about 50% of normal adult levels [3]. Newborn from mothers with vitamin K deficiency may be more susceptible to intraventricular hemorrhage.

Vitamin K deficiency in our patient was associated with intrahepatic cholestasis. However, whether the vitamin K deficiency was caused by intrahepatic cholestasis or nutritional deficiency or both cannot be determined. Presenting with no prenatal care is a clue that she may not have had social support. Her low BMI suggests a potential nutritional concern. She apparently did not comply with or have the ability to fill prescriptions. In retrospect, we believe that Ursodeoxycholic acid therapy may have been started sooner to alleviate her pruritic symptoms, although some may argue that there is a theoretical risk that Ursodeoxycholic acid may reduce the absorption of vitamin K. Indomethacin was used cautiously because of her compromised hepatic function.

Use and absorption of oral vitamin K are not well understood [6]. A 1982 study showed low levels of maternal to fetal transfer after IV administration of vitamin K prior to delivery [4]. The authors suggested a potential beneficial effect of this therapy [3, 6] although it has not been adopted. Prophylactic administration of vitamin K to the neonate is recommended [5].

In developing countries, obstetric hemorrhage is the leading direct cause of both maternal mortality and near-miss events [1]. Mainly due to severe obstetrical hemorrhage, the immediate postpartum period is associated with 60.6% of maternal deaths compared to 23.9% and 15.5% in antepartum and intrapartum period [7], making the immediate postpartum period associated with the highest risk for maternal mortality.

In summary, patients with atypical bleeding in pregnancy such as hematuria or presenting symptoms suggestive of ICP (pruritus in our case) should be evaluated for possible vitamin K deficiency related to ICP. Patients with ICP should have a coagulation profile checked when admitted for labor or preoperatively for patients delivered by scheduled cesarean section. Coagulation abnormalities from vitamin K deficiency can be corrected immediately with FFP which normalizes the INR. The treatment therapy should be continued with Ursodeoxycholic acid and vitamin K, in order to prevent maternal and neonatal morbidity and mortality postpartum.

## Figures and Tables

**Table 1:** Laboratory values at initial presentation.

Laboratory test	Result
Platelets	196 K/mL
Prothrombin Time (PT)	117.8 seconds
Activated Partial Thromboplastin Time (aPTT)	80.5 seconds
International Normalized Ratio (INR)	10.34
Fibrinogen	622 mg/dL
Total bile acids	29 *µ*/L
AST	72 units/L
ALT	68 units/L

## References

[1] Tanimia H., Jayaratnam S., Mola G. L., Amoa A. B., De Costa C. (2016). Near-misses at the Port Moresby General Hospital: A descriptive study. *Australian and New Zealand Journal of Obstetrics and Gynaecology*.

[2] Lane P. A., Hathaway W. E. (1985). Vitamin K in infancy. *The Journal of Pediatrics*.

[3] Pichler E., Pichler L. (2008). The neonatal coagulation system and the vitamin K deficiency bleeding - A mini review. *Wiener Medizinische Wochenschrift*.

[4] Shearer M. J., Barkhan P., Rahim S., Stimmler L. (1982). Plasma vitamin k1 in mothers and their newborn babies. *The Lancet*.

[5] Sadler L. C., Lane M., North R. (1995). Severe fetal intracranial haemorrhage during treatment with cholestyramine for intrahepatic cholestasis of pregnancy. *BJOG: An International Journal of Obstetrics & Gynaecology*.

[6] Minami H., Furuhashi M., Minami K., Miyazaki K., Yoshida K., Ishikawa K. (2009). Fetal intraventricular bleeding possibly due to maternal vitamin K deficiency. *Fetal Diagnosis and Therapy*.

[7] Thaddeus S., Nangalia R., Vivio D. (2004). Perceptions matter: Barriers to treatment of postpartum hemorrhage. *Journal of Midwifery and Women's Health*.

